# Author Correction: Growth hormone regulates neuroendocrine responses to weight loss via AgRP neurons

**DOI:** 10.1038/s41467-019-09022-2

**Published:** 2019-02-25

**Authors:** Isadora C. Furigo, Pryscila D. S. Teixeira, Gabriel O. de Souza, Gisele C. L. Couto, Guadalupe García Romero, Mario Perelló, Renata Frazão, Lucila L. Elias, Martin Metzger, Edward O. List, John J. Kopchick, J. Donato

**Affiliations:** 1 Department of Physiology and Biophysics, Institute of Biomedical Sciences, University of São Paulo, Av. Prof. Lineu Prestes, 1524, São Paulo, SP 05508-000 Brazil; 2 Laboratory of Neurophysiology, Multidisciplinary Institute of Cell Biology, Calle 526 y Camino General Belgrano, La Plata, BA 1900 Argentina; 3 Department of Anatomy, Institute of Biomedical Sciences, University of São Paulo, Av. Prof. Lineu Prestes, 2415, São Paulo, SP 05508-900 Brazil; 4 Department of Physiology, School of Medicine of Ribeirão Preto, University of São Paulo, Av. Bandeirantes, 3900, Ribeirão Preto, SP 14049-900 Brazil; 5 Edison Biotechnology Institute and Heritage College of Osteopathic Medicine, Ohio University, Konneker Research Center 206A, Athens, OH 45701 USA

Correction to: *Nature Communications;* 10.1038/s41467-019-08607-1; published online 08 February 2019

The original version of this Article contained an error in the spelling of the author J. Donato Jr, which was incorrectly given as Donato J. Jr. This has now been corrected in both the PDF and HTML versions of the Article.

