# Supplementary material for: Growth hormone regulates neuroendocrine responses to weight loss via AgRP neurons
Source: Nat Commun. 2019 Feb 8;10:662. doi: 10.1038/s41467-019-08607-1 (PMC6368581; doi:10.1038/s41467-019-08607-1)
Supplement: Supplementary file 1 — Supplementary Information [file 41467_2019_8607_MOESM1_ESM.pdf]

Growth hormone regulates neuroendocrine responses to weight loss via AgRP neurons

Furigo et al.

Supplementary Figures

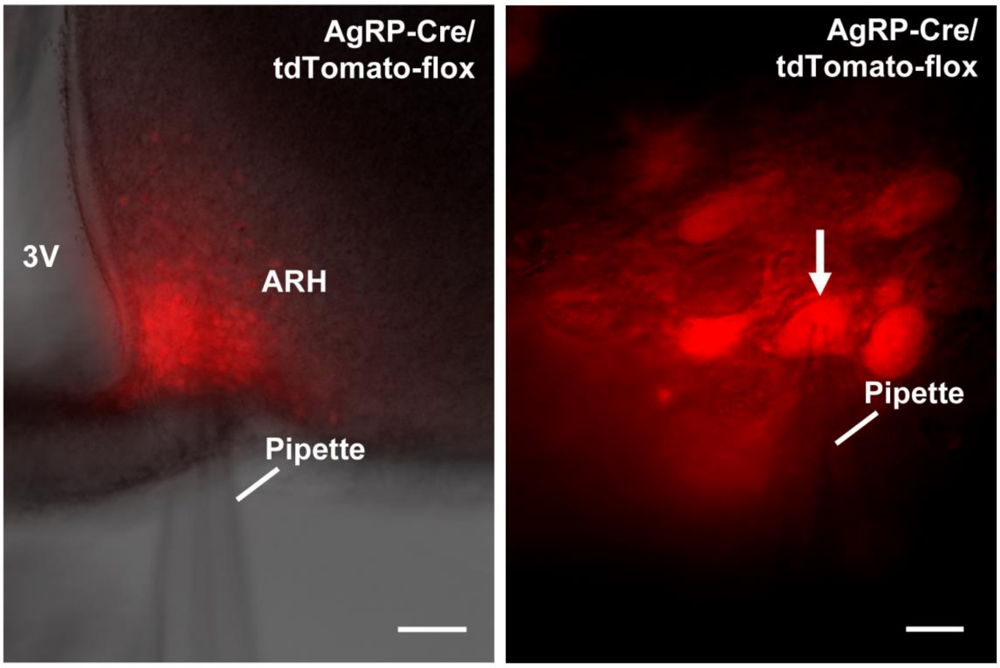

Supplementary Figure 1. Whole-cell patch-clamp recording in AgRP neurons of the arcuate nucleus. Low-(left) and high-(right) power images showing a recorded AgRP fluorescent neuron contacted with the pipette (indicated by arrow). Abbreviations: 3V, third ventricle; ARH, arcuate nucleus. Scale bars = 75 µm (left) and 10 µm (right).

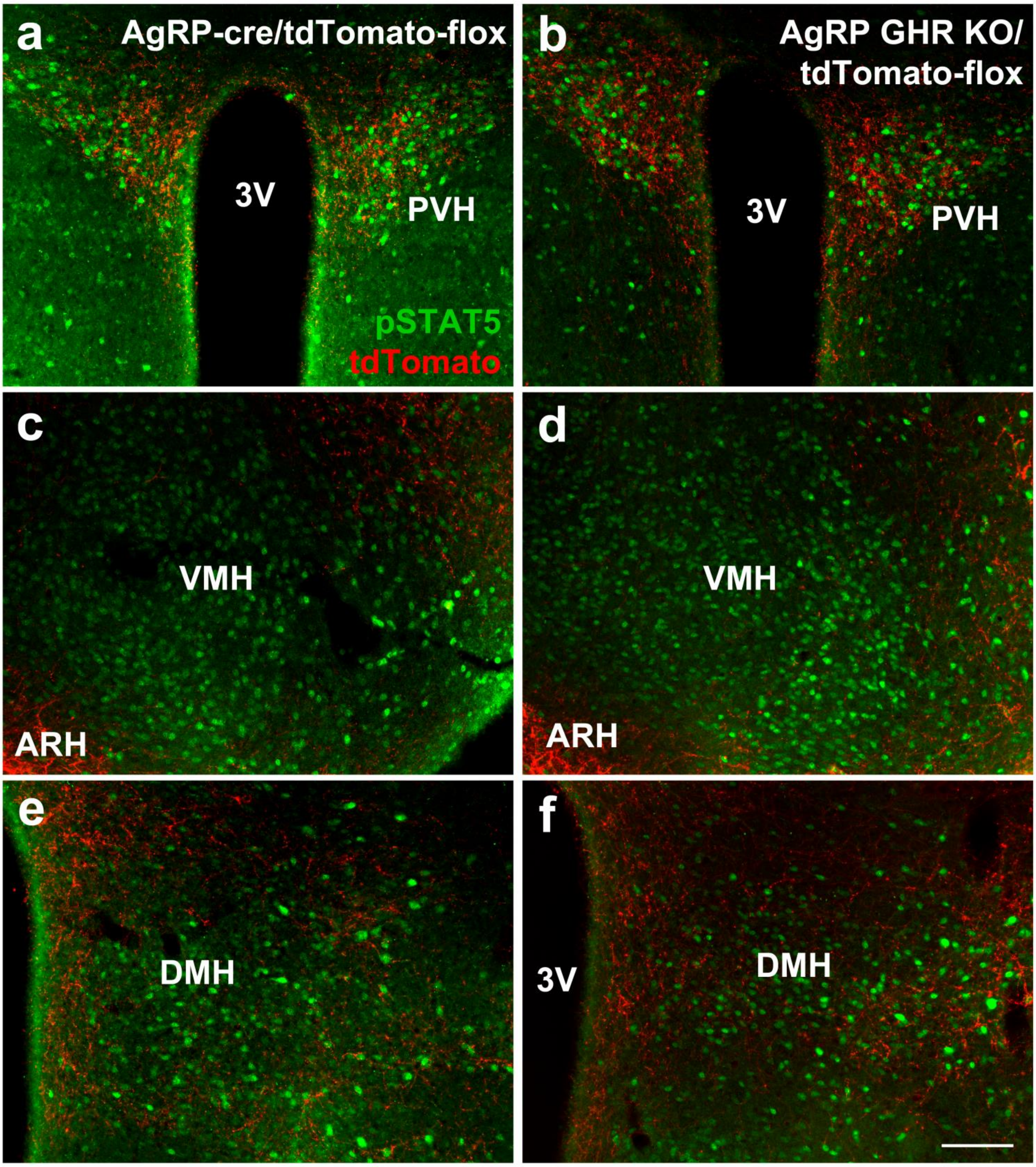

Supplementary Figure 2. Distribution of GH responsive cells in several hypothalamic nuclei of control and AgRP GHR KO mice. a-f, Epifluorescence photomicrographs showing the distribution of AgRP fibers (red) and STAT5 phosphorylation (pSTAT5; green) 90 min after an i.p. injection of porcine GH (20 µg/g) in AgRP-Cre/tdTomato-flox mice (control) and AgRP GHR KO/tdTomato-flox mice. Abbreviations: 3V, third ventricle; ARH, arcuate nucleus; DMH, dorsomedial nucleus; PVH, paraventricular nucleus; VMH, ventromedial nucleus. Scale Bar = 100 µm.

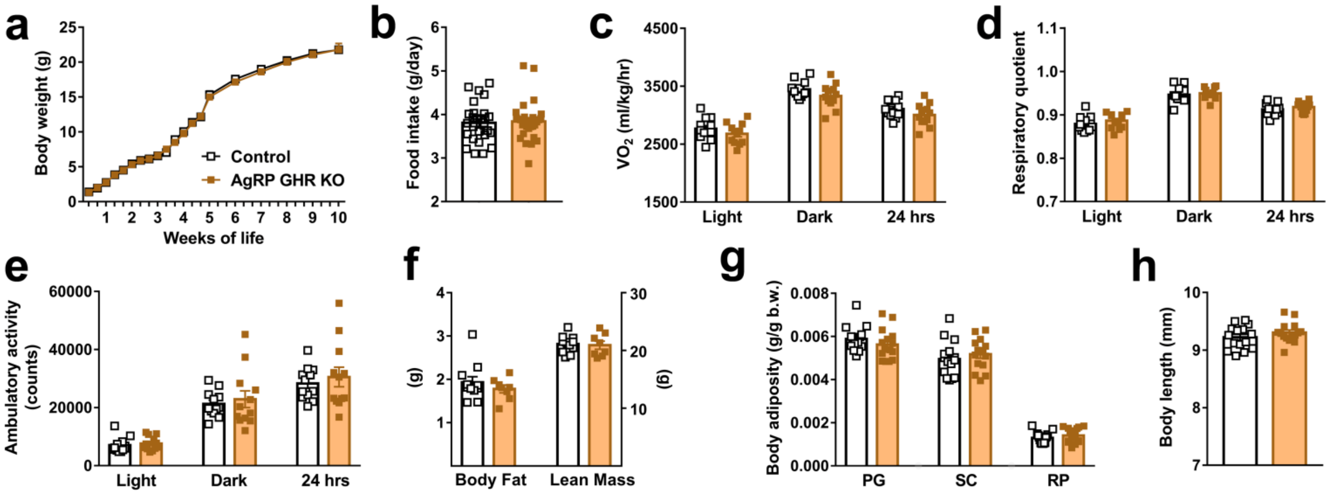

Supplementary Figure 3. AgRP GHR KO mice showed no evidence of metabolic imbalances. a, Body weight changes in control and AgRP GHR KO mice (*n* = 8-19 depending on age). **b**, Daily food intake (*n* = 27-28) of 5 month old male mice. **c-e**, Energy expenditure (VO_2), respiratory quotient and ambulatory activity in the light, dark and 24 h cycles of 6 month old male mice (*n* = 12). **f**, Body composition analysis of 6 month old control (*n* = 11) and AgRP GHR KO (*n* = 8) male mice. **g**, The masses of the perigonadal (PG), subcutaneous (SC) and retroperitoneal (RP) fat pads of 6 month old male mice (*n* = 15). **h**, Body length of 6 month old male mice (*n* = 15). The unpaired two-tailed Student's *t*-test was used for comparisons. Mean ± s.e.m.

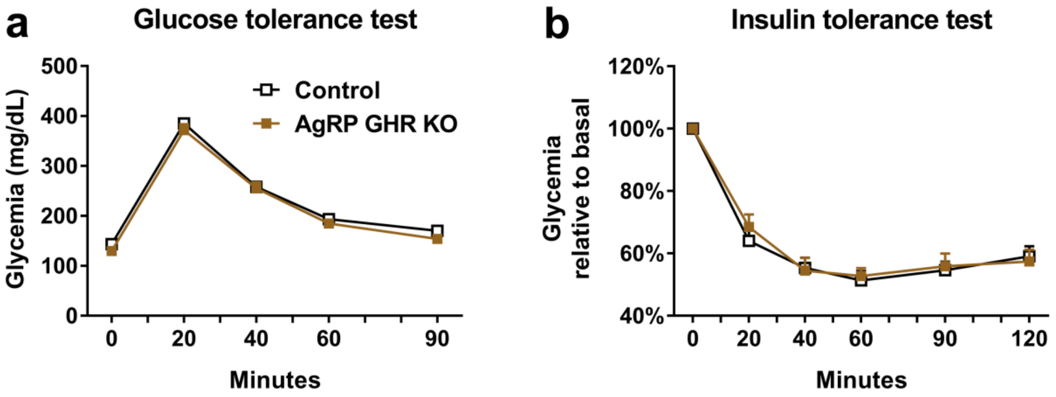

Supplementary Figure 4. Normal glucose and insulin tolerance in AgRP GHR KO mice. a-b, Blood glucose levels in 5 month old control and AgRP GHR KO male mice during i.p. infusions of glucose (2 g/kg b.w; *n* = 26-27) or insulin (1 IU/kg b.w.; *n* = 12). Mean ± s.e.m.

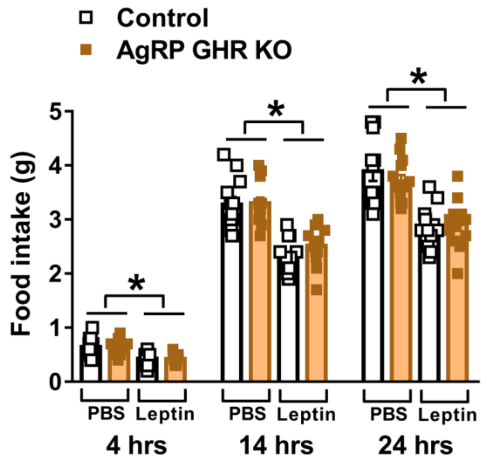

Supplementary Figure 5. Normal leptin sensitivity in AgRP GHR KO mice. Five month old control and AgRP GHR KO male mice (*n* = 12-13) received i.p. injections of either phosphate-buffered saline (PBS) or mouse recombinant leptin (2.5 µg/g b.w.) 3 h before their dark phase, and their food intake were recorded 4 h (main effect of leptin [$F_{(1, 23)} = 34.64$, $P < 0.0001$], main effect of GHR ablation [$F_{(1, 23)} = 0.3196$, $P = 0.5773$] and interaction [$F_{(1, 23)} = 0.305$, $P = 0.5861$]), 14 h (main effect of leptin [$F_{(1, 23)} = 97.74$, $P < 0.0001$], main effect of GHR ablation [$F_{(1, 23)} = 0.7764$, $P = 0.3874$] and interaction [$F_{(1, 23)} = 1.056$, $P = 0.3148$]) and 24 h (main effect of leptin [$F_{(1, 23)} = 64.68$, $P < 0.0001$], main effect of GHR ablation [$F_{(1, 23)} = 0.1799$, $P = 0.6754$] and interaction [$F_{(1, 23)} = 1.127$, $P = 0.2994$]) following the injection. The data were analyzed by Two-way ANOVA and Newman-Keuls post-hoc tests. Mean ± s.e.m.

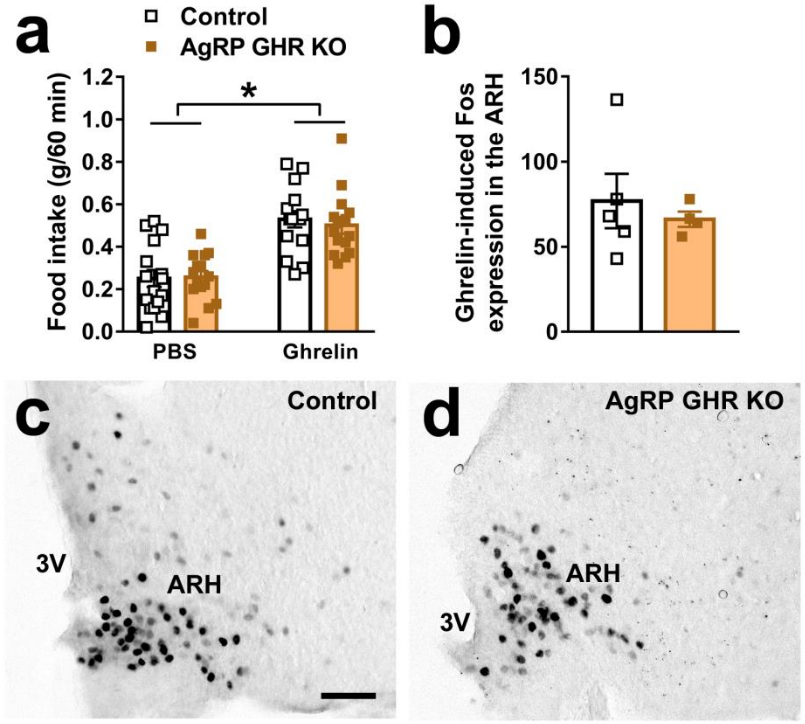

Supplementary Figure 6. Normal orexigenic response to ghrelin in AgRP GHR KO mice. a, Control and AgRP GHR KO mice (*n* = 15-16) received subcutaneous injections of either PBS or ghrelin (0.2 µg/g b.w.), and their food intake was assessed 60 min afterwards (main effect of ghrelin [$F_{(1, 29)}$ = 56.7, $P < 0.0001$], main effect of GHR ablation [$F_{(1, 29)}$ = 0.0743, $P = 0.787$] and interaction [$F_{(1, 29)}$ = 0.2505, $P = 0.6205$]; Two-way ANOVA and Newman-Keuls post-hoc test). **b**, The number of c-Fos positive cells in the arcuate nucleus of the hypothalamus (ARH) 90 min after an acute subcutaneous ghrelin infusion (0.2 µg/g b.w.; $t_{(7)}$ = 0.581, $P = 0.5795$, *n* = 4-5; unpaired *t* test). **c-d**, Photomicrographs showing ghrelin-induced c-Fos expression in the ARH of control and AgRP GHR KO mice. 3V, third ventricle. Scale Bar = 50 µm. Mean ± s.e.m.

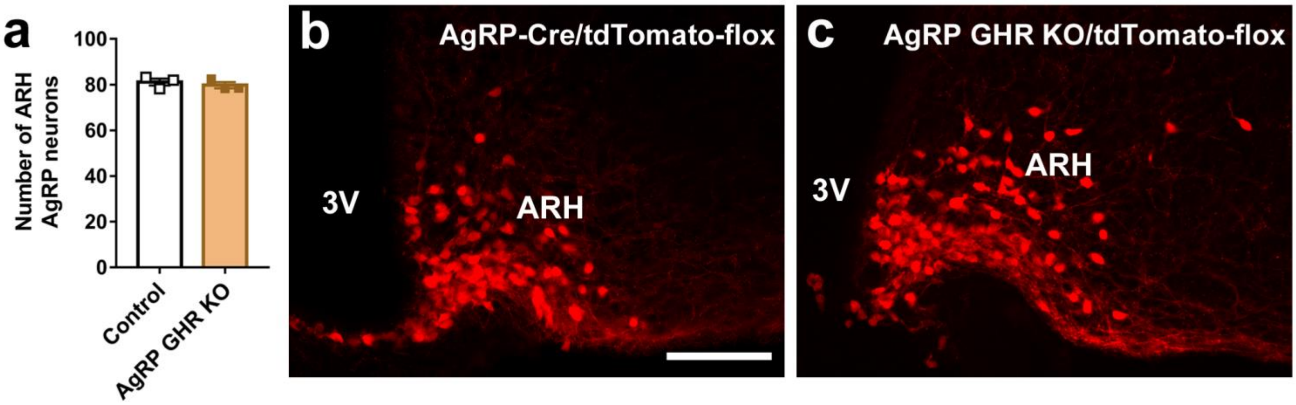

Supplementary Figure 7. No changes in the number of AgRP neurons in the ARH of AgRP GHR KO mice. a-c, The reporter tdTomato fluorescent protein was used to determine the number of AgRP neurons in the ARH of control and AgRP GHR KO mice ($t_{(4)}$ = 0.6849, P = 0.531, n = 3; unpaired t test). Mean ± s.e.m. 3V, third ventricle. Scale Bar = 100 µm.

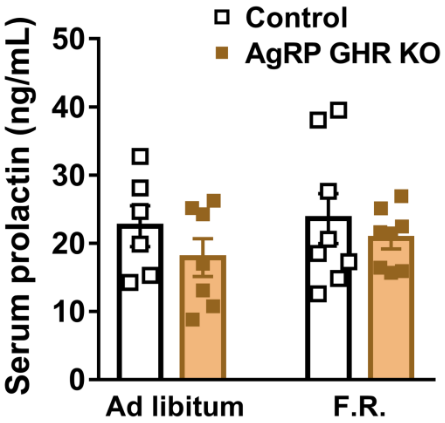

Supplementary Figure 8. Serum prolactin concentration in 6 month old male mice with *ad libitum* access to food or during food restriction. Main effect of food restriction [$F_{(1, 25)}$ = 0.4697, *P* = 0.4994], main effect of GHR ablation [$F_{(1, 25)}$ = 1.729, *P* = 0.2005] and interaction [$F_{(1, 25)}$ = 0.0852, *P* = 0.7727]; Two-way ANOVA. Mean ± s.e.m.

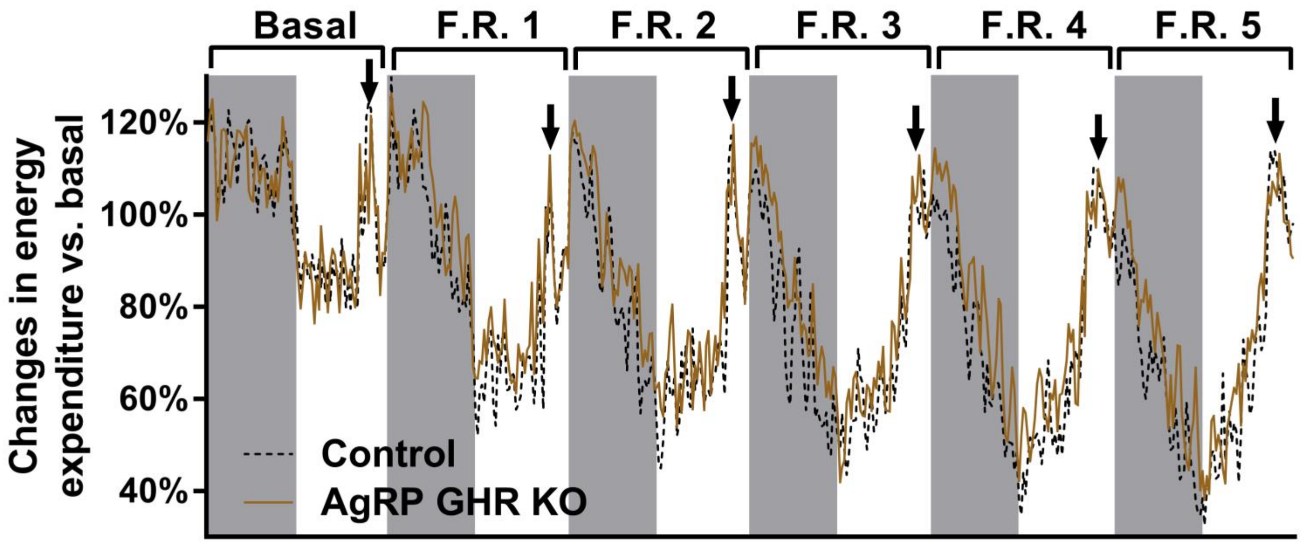

Supplementary Figure 9. Changes in energy expenditure (VO_2) in the baseline and during food restriction. Each mouse received 40% of their normal intake 2 h before lights off (indicated by arrows) for 5 consecutive days. Note that during the basal state, control and AgRP GHR KO mice exhibited a very similar energy expenditure. However, during food restriction the energy expenditure of AgRP GHR KO mice remained above the values of the control group during most of the time. Gray and white backgrounds represent dark and light cycles, respectively. This figure shows the mean values of each group (*n* = 13-14).

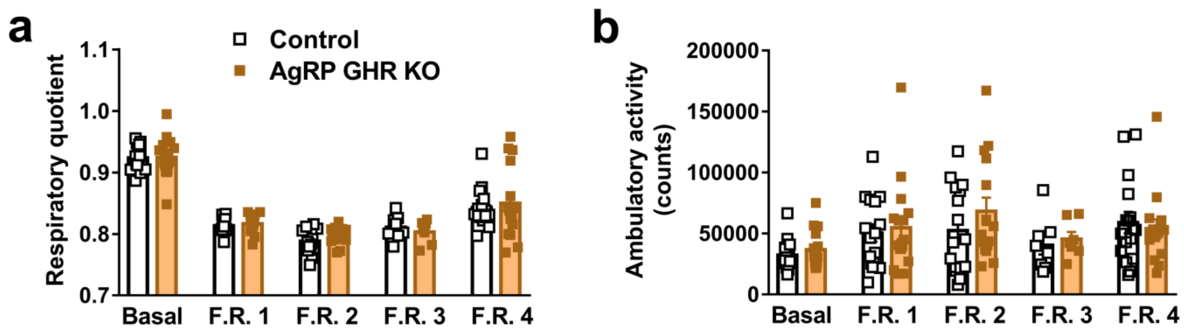

Supplementary Figure 10. Respiratory quotient and ambulatory activity during food restriction. a-b, Changes in the respiratory quotient and in ambulatory activity of control and AgRP GHR KO mice in the basal state and during 60% food restriction (*n* = 13-14). Mean ± s.e.m.

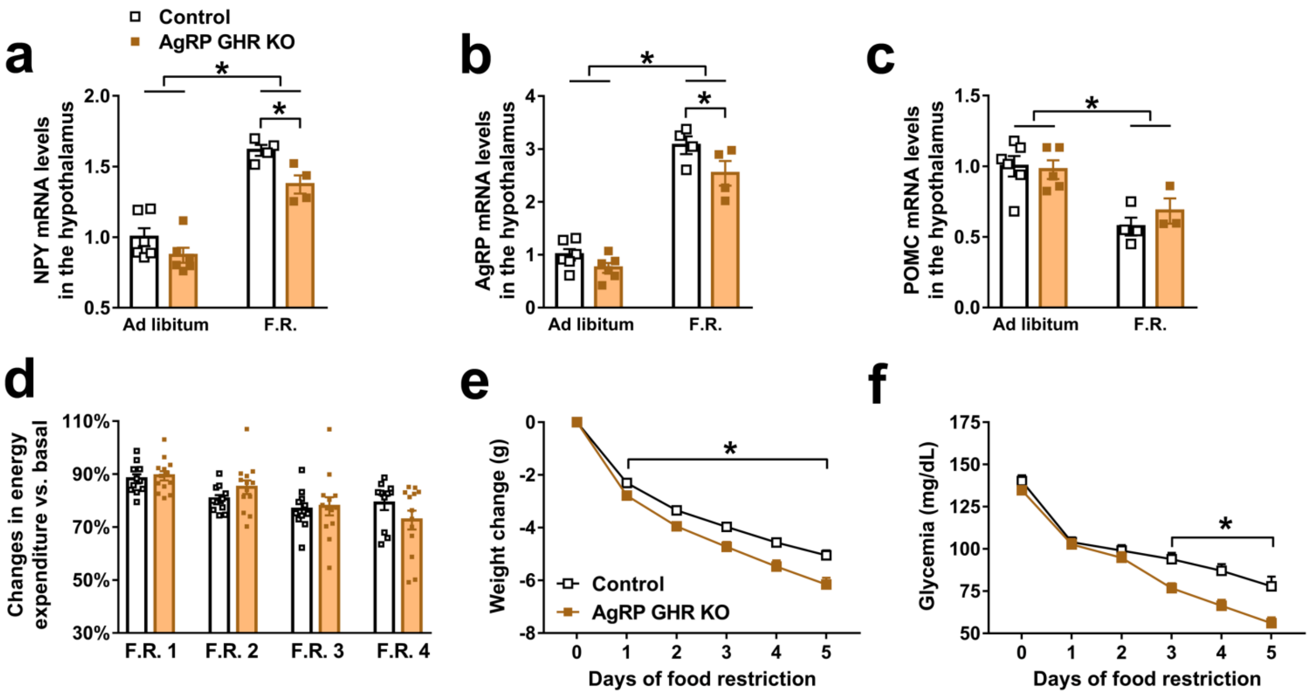

Supplementary Figure 11. Neuroendocrine changes induced by weight loss in AgRP GHR KO females. a-b, Two days of 60% of food restriction (F.R.; 40% of the normal intake) changed hypothalamic mRNA expression of NPY (main effect of F.R. [$F_{(1, 16)}$ = 86.8, *P* < 0.0001], main effect of GHR ablation [$F_{(1, 16)}$ = 9.543, *P* = 0.007] and interaction [$F_{(1, 16)}$ = 0.9038, *P* = 0.3559]) and AgRP (main effect of F.R. [$F_{(1, 16)}$ = 177.6, p < 0.0001], main effect of GHR ablation [$F_{(1, 16)}$ = 7.239, *P* = 0.0161] and interaction [$F_{(1, 16)}$ = 0.9541, *P* = 0.3432]) in control females, but these effects were attenuated in AgRP GHR KO female mice (*n* = 4-6). **c**, Hypothalamic mRNA expression of POMC (main effect of F.R. [$F_{(1, 14)}$ = 22.37, *P* = 0.0003], main effect of GHR ablation [$F_{(1, 14)}$ = 0.3248, *P* = 0.5778] and interaction [$F_{(1, 14)}$ = 0.7733, *P* = 0.3940]; *n* = 3-6). **d,** Reduction in energy expenditure (VO_2) during the days of F.R. compared to baseline (F.R. 1, $t_{(22)}$ = 0.4509, *P* = 0.6565; F.R. 2, $t_{(22)}$ = 1.421, *P* = 0.1693; F.R. 3, $t_{(22)}$ = 0.2595, *P* = 0.7977; F.R. 4, $t_{(22)}$ = 1.396, *P* = 0.1766; *n* = 11-13; unpaired *t* test). **e-f**, Changes in body weight (main effect of F.R. [$F_{(5, 310)}$ = 931.7, *P* < 0.0001], main effect of GHR ablation [$F_{(1, 62)}$ = 13.07, *P* = 0.0006] and interaction [$F_{(5, 310)}$ = 8.401, *P* < 0.0001]; *n* = 31-33) and glycemia (main effect of F.R. [$F_{(5, 310)}$ = 144.1, *P* < 0.0001], main effect of GHR ablation [$F_{(1, 62)}$ = 12.9, *P* = 0.0006] and interaction [$F_{(5, 310)}$ = 4.861, *P* = 0.0003]; *n* = 31-33) during 60% F.R. Mean ± s.e.m.

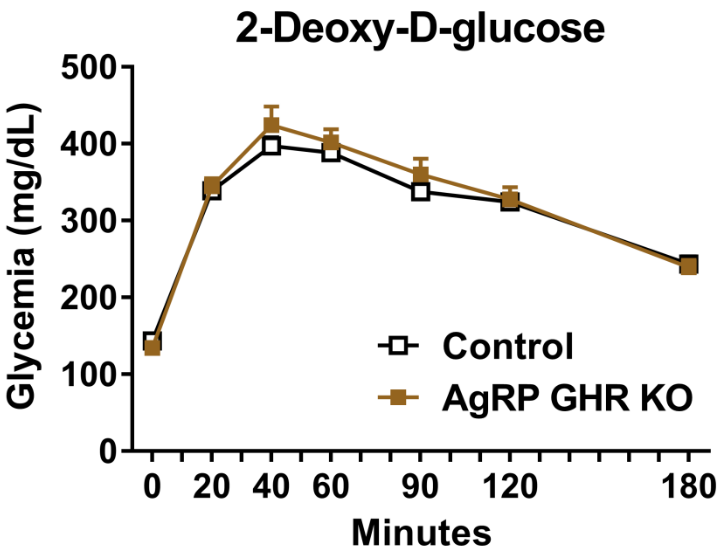

Supplementary Figure 12. Counter-regulatory response induced by 2-Deoxy-D-glucose (2DG) infusion. Control (*n* = 12) and AgRP GHR KO (*n* = 12) mice received an i.p. injection of 2DG (0.5 mg/kg b.w.) and their tail blood glucose levels were measured during 180 min. Data were analyzed by two-way ANOVA (main effect of time [$F_{(6, 132)}$ = 197.6, $P < 0.0001$], main effect of GHR ablation [$F_{(1, 22)}$ = 0.3254, $P = 0.5742$] and interaction [$F_{(6, 132)}$ = 0.9669, $P = 0.4503$]) and Fisher LSD post-hoc test. Mean ± s.e.m.

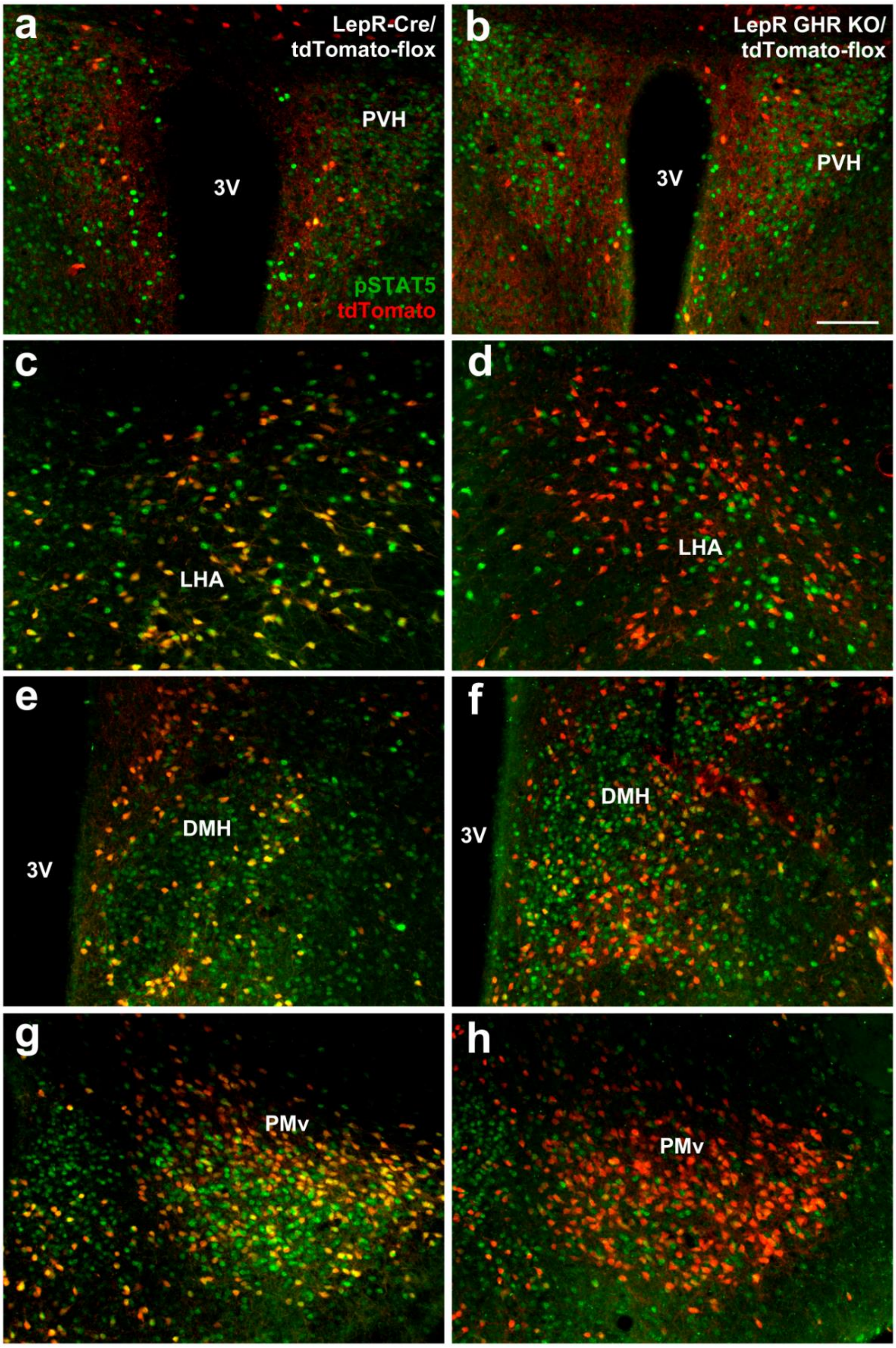

Supplementary Figure 13. Distribution of GH responsive cells in several hypothalamic nuclei of control and LepR GHR KO mice. a-h, Epifluorescence photomicrographs showing the distribution of LepR-expressing cells (red) and STAT5 phosphorylation (pSTAT5; green) 90 min after an i.p. injection of porcine GH (20 µg/g) in LepR-IRES-Cre/tdTomato-flox mice (control) and LepR GHR KO/tdTomato-flox mice. Yellow represents double-labeled cells. Abbreviations: 3V, third ventricle; DMH, dorsomedial nucleus; LHA, lateral hypothalamic area; PMv, ventral premammillary nucleus; PVH, paraventricular nucleus; VMH, ventromedial nucleus. Scale Bar = 100 µm.

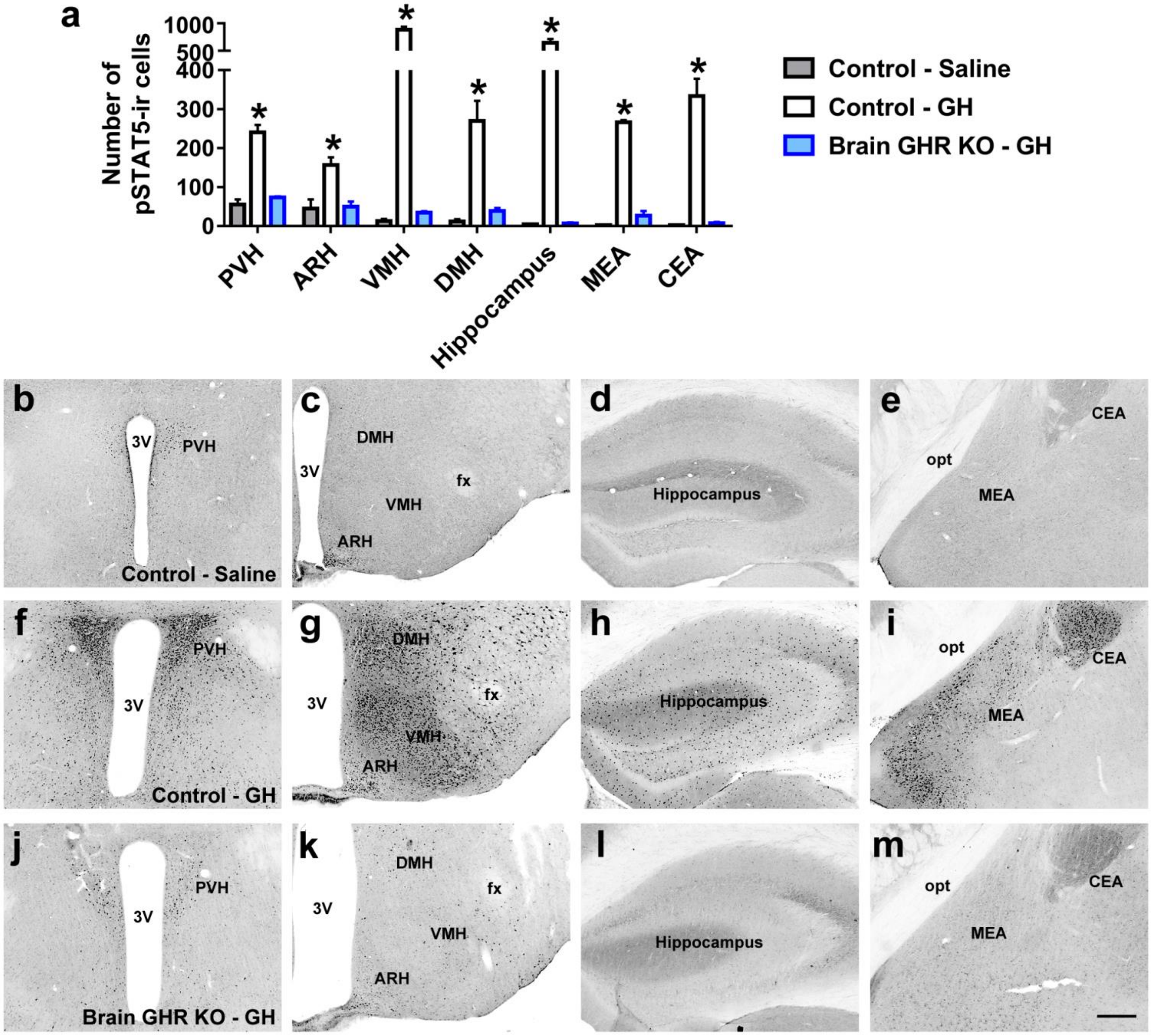

Supplementary Figure 14. Confirmation of brain-specific GHR deletion. **a**, Number of cells expressing pSTAT5-immunoreactivity (pSTAT5-ir) in different brain nuclei (*n* = 3/group). Mice were perfused 90 min after an i.p. injection of saline or porcine GH (20 µg/g b.w.). Cells were counted in one rostrocaudal level of each area using the ImageJ software. Data were analyzed using one-way ANOVA and the Newman-Keuls multiple comparison tests (PVH: $F_{(2, 6)} = 54.37$, $P = 0.0001$; ARH: $F_{(2, 6)} = 10.35$, $P = 0.0114$; VMH: $F_{(2, 6)} = 258.8$, $P < 0.0001$; DMH: $F_{(2, 6)} = 21.72$, $P = 0.0018$; hippocampus: $F_{(2, 6)} = 77.98$, $P < 0.0001$; MEA: $F_{(2, 6)} = 349$, $P < 0.0001$; CEA: $F_{(2, 6)} = 52.85$, $P = 0.0002$). Mean ± s.e.m. **b-m**, Brightfield photomicrographs of mouse brain sections showing the distribution of pSTAT5 immunoreactive cells in saline-injected control mice (b-e), GH-injected control mice (f-i) and GH-injected brain GHR KO mice (j-m). Abbreviations: 3V, third ventricle; ARH, arcuate nucleus; CEA, central nucleus of the amygdala; DMH, dorsomedial nucleus; fx, fornix; MEA, medial nucleus of the amygdala; opt, optic tract; PVH, paraventricular nucleus; VMH, ventromedial. Scale Bar = 200 µm.

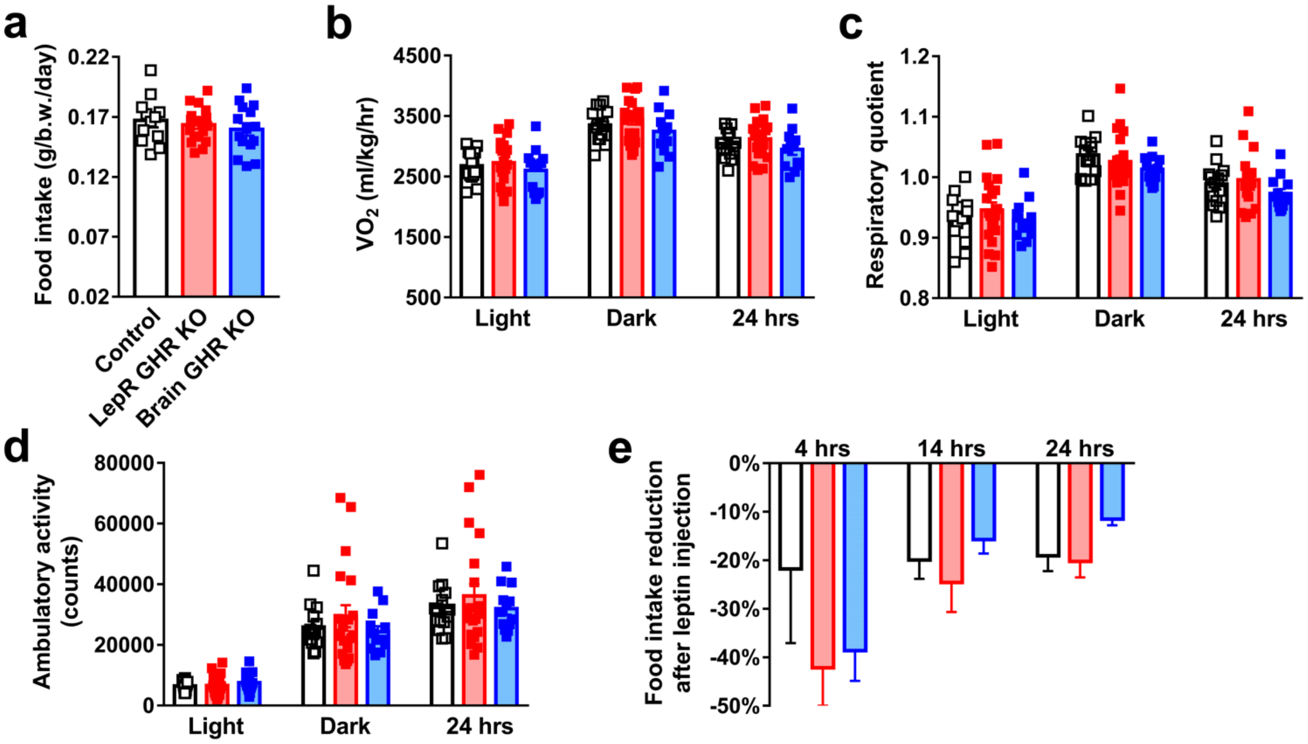

Supplementary Figure 15. Metabolic aspects of LepR GHR KO and Brain GHR KO mice. a-d, Daily food intake, energy expenditure (VO_2), respiratory quotient and ambulatory activity in the light, dark and 24 h cycles (n = 11-20/group). **e**, Anorexigenic response to leptin in control, LepR GHR KO and Brain GHR KO mice that received i.p. injections of either PBS or mouse recombinant leptin (2.5 μg/g b.w.; n = 5-8/group) 3 h before their dark phase. Food intake after PBS injection was compared with food intake after leptin administration. One-way ANOVA and the Newman-Keuls multiple comparison tests were used for the comparisons. Mean ± s.e.m.

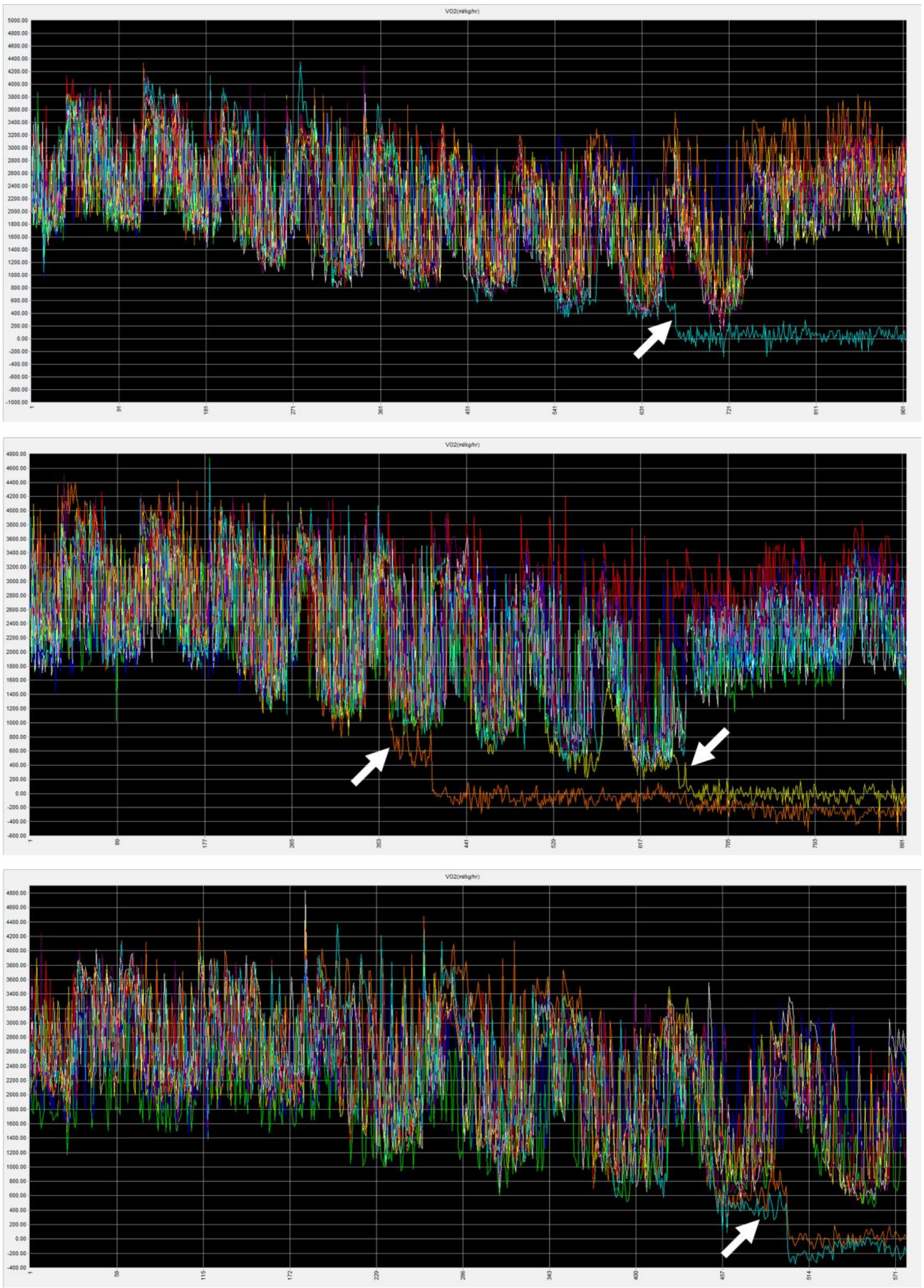

Supplementary Figure 16. Severe decline of the energy expenditure in several LepR GHR KO mice. Each of these figures illustrates the changes in energy expenditure (VO_2) of 8 mice before and during 60% of food restriction (40% of the normal intake). From 24 mice analyzed in this experiment, 11 were from the LepR GHR KO group. The arrows indicate that 5 mice (45%) from the LepR GHR KO group exhibited a sharp decline in the oxygen consumption between the third and fifth day of food restriction (their energy expenditure dropped to about one-sixth of the initial value). These mice became lethargic during food restriction and had to be euthanized and removed from the study.
